# Long-term follow-up of patients with ATL after autologous stem cell transplantation

**DOI:** 10.1038/s41409-021-01412-9

**Published:** 2022-01-23

**Authors:** Atae Utsunomiya, Masahito Tokunaga, Nobuaki Nakano, Hiroshi Fujiwara, Toshihiro Miyamoto, Masao Ogata, Yasuhiko Miyazaki, Kenji Ishitsuka, Emiko Sakaida, Hirofumi Taji, Toshio Wakayama, Tatsuo Ichinohe, Takahiro Fukuda, Yoshiko Atsuta, Koji Kato, Makoto Yoshimitsu

**Affiliations:** 1 Department of Hematology, Imamura General Hospital, Kagoshima, Japan; 2 Department of Hematology, Mie University Hospital, Tsu, Japan; 3 Department of Medicine and Biosystemic Science, Kyushu University Graduate School of Medicine, Fukuoka, Japan; 4 Department of Hematology, Oita University Hospital, Oita, Japan; 5 Department of Hematology, Oita Prefectural Hospital, Oita, Japan; 6 Department of Hematology and Rheumatology, Kagoshima University Hospital, Kagoshima, Japan; 7 Department of Hematology, Chiba University Hospital, Chiba, Japan; 8 Department of Hematology and Cell Therapy, Aichi Cancer Center Hospital, Nagoya, Japan; 9 Department of Hematology and Oncology, Shimane Prefectural Central Hospital, Izumo, Japan; 10 Department of Hematology and Oncology, Research Institute for Radiation Biology and Medicine, Hiroshima University, Hiroshima, Japan; 11 Department of Hematopoietic Stem Cell Transplantation, National Cancer Center Hospital, Tokyo, Japan; 12 Japanese Data Center for Hematopoietic Cell Transplantation, Nagoya, Japan; 13 Department of Registry Science for Transplant and Cellular Therapy, Aichi Medical University School of Medicine, Nagakute, Japan

**Keywords:** T-cell lymphoma, Acute myeloid leukaemia

## To the Editor:

Adult T-cell leukemia/lymphoma (ATL) is a refractory peripheral T-cell lymphoma (PTCL). Treatment with chemotherapy alone is associated with an extremely poor prognosis. Allogeneic stem cell transplantation (SCT) is the only modality shown to achieve long-term survival, but treatment-related mortality is a critical problem in the relatively elderly individuals most likely to develop with ATL [1]. Autologous stem cell transplantation (ASCT) is rarely performed in ATL, and the few studies of ASCT in ATL reported dismal outcomes [2–4].

To assess the efficacy of ASCT as a means of reducing tumor burden in the era of autologous immune activation therapies, such as immune checkpoint inhibitors [5], this study was performed to characterize the status of autografting in ATL using national registry data in Japan, one of the most endemic areas for ATL.

The clinical data of hematopoietic SCT recipients were collected by the Japan Society for Hematopoietic Cell Transplantation (JSHCT) and the Japanese Data Center for Hematopoietic Cell Transplantation using the Transplant Registry Unified Management Program (TRUMP) [6]. Data were maintained by the JSHCT (TRUMP database, last update, December 2018).

A total of 2216 patients with ATL who underwent SCT between 1991 and 2017 were registered in the TRUMP database. Twenty-seven patients underwent ASCT, one received syngeneic SCT, and 2188 were treated with allogeneic SCT. The 27 ASCT patients were retrospectively analyzed. Demographic characteristics, ATL subtypes, stem cell sources, preconditioning regimens, disease status at ASCT, ECOG performance status at ASCT, date at diagnosis, date at ASCT, date at neutrophil engraftment, date at last observation, and causes of death were collected. Overall survival (OS) was calculated from the date of ASCT and diagnosis until the date of death or final follow-up.

This study was approved by the data management committees of JSHCT and the institutional ethical committee of Imamura General Hospital.

OS was analyzed using the Kaplan–Meier method. In four patients who underwent SCT twice, the date of the second transplant was not censored. Univariate comparisons of OS were made using the log rank test. *P* values < 0.05 were considered statistically significant. Statistical analyses were performed using EZR software [7].

Patient characteristics are shown in Supplementary Table 1. Patients consisted of 14 males and 13 females, with a median age at ASCT of 52 years (range, 31–66 years). Twenty-four of 27 patients (88.9%) received ASCT in or before 2009. ASCT was performed in 11 patients (40.7%) with complete remission (CR), three (11.1%) with relapse, and 13 (48.1%) with primary induction failure. Eleven patients had aggressive-type ATL, including five with acute type and six with lymphoma type, whereas 16 patients had an unknown subtype. The Eastern Cooperative Oncology Group performance status was 0 in four patients, 1 in seven, and unknown in 16. Peripheral blood stem cells were used as the stem cell source in 24 patients (88.5%), while three patients received autologous bone marrow transplantation. The median duration from diagnosis to ASCT was 242.5 days (range, 64–1128 days). Four patients received SCT twice; of these, one underwent ASCT twice and the other three underwent ASCT followed by allogeneic SCT (Supplementary Table 2).

Data on preconditioning regimens were available for only eight patients. Cyclophosphamide was used in five patients, etoposide in six, ranimustine in five, carboplatin in two, cytosine arabinoside in five, melphalan in three, ifosfamide in one, and dexamethasone in two. Only one patient underwent total body irradiation as preconditioning therapy.

**Fig. 1:**
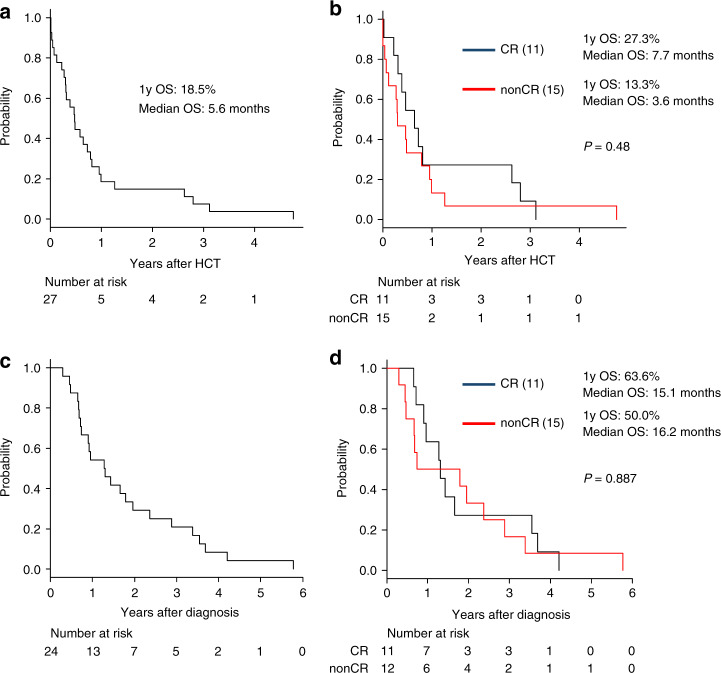
Overall survival from ASCT and from diagnosis. **a** Overall survival from ASCT in patients with ATL who underwent ASCT, (**b**) Overall survival according to CR or nonCR, (**c**) Overall survival from diagnosis, (**d**) Overall survival according to remission state. OS overall survival, ASCT autologous stem cell transplantation, CR complete remission, Diag diagnosis.

Data on neutrophil engraftment were obtained for 25 patients (92.6%). Two patients died before engraftment. The 1-year OS rate after ASCT was 18.5%, and the median OS time (MST) was 5.6 months (Fig. 1a). The 1-year OS rate and the MST were 27.3% and 7.7 months, respectively, in patients with CR at ASCT, and 13.3% and 3.6 months, respectively, in patients without CR (Fig. 1b). The 3-year OS rate and the MST after diagnosis were 20.8% and 15.5 months, respectively. The MST was 15.1 and 16.2 months in patients with CR and without CR, respectively (Fig. 1c, d). All 27 patients who underwent ASCT died during the observation period. The causes of death were ATL in 15 patients, infections in six, and unknown in six.

All four patients who underwent SCT twice died, with OS ranging from 3 to 11 months. The causes of death were pneumonia in three patients and ATL in one (Supplementary Table 2).

This study is the first descriptive case series to summarize the results of ASCT in patients with ATL. Patients with ATL who underwent ASCT had poor outcomes, with a 1-year OS rate of 18.5% and a MST of 5.6 months. Four patients survived for longer than 30 months after ASCT, and it should be noted that this duration of survival after ASCT has not been reported previously. Although the overall response to treatment remains poor, this study provides more accurate data on long-term outcomes since it used information from a registry that requires registration of almost all patients receiving SCT. The study also revealed that autologous transplants have rarely been performed in Japan since 2010.

Among patients with a known cause of death, 71.4% died of ATL relapse, and the remaining 28.6% died of infection. In a previous report by Tsukasaki et al., the causes of death of eight patients were recurrence of primary ATL in four, pneumonia in one, and unknown in three [2]. The percentage of deaths due to infection in this study was 28.6%; this result differed markedly from that based on Japanese registry data, which showed that only 2% of patients with PTCL-not otherwise specified who underwent ASCT died of infection [8]. This study therefore identified infection as a crucial contributor to treatment-related mortality following ASCT for ATL. This should be kept in mind when developing new therapies involving ASCT.

Most patients in this study underwent autologous transplants before 2010. Therefore, new molecular targeting drugs such as mogamulizumab, lenalidomide, and brentuximab vedotin were not used. In some cases, mogamulizumab can promptly eradicate CCR4-positive peripheral ATL lesions, which may allow for remission induction therapy with only low-intensity chemotherapy to reduce the initial tumor volume, and mogamulizumab may also reduce the contamination of autologous grafts with ATL cells during the collection of peripheral blood hematopoietic stem cells [9]. Therefore, autologous transplantation after remission induction therapy with mogamulizumab may reduce the risk of ATL relapse and the rate of complications due to infection. A new immunotherapy, the Tax peptide-pulsed dendritic cell vaccine, demonstrated good outcomes in patients with aggressive ATL who were in stable condition [10]. This approach may be another viable option to extend the applicability of ASCT for ATL patients who are ineligible for allogeneic SCT.

The limitations of this study include the small number of patients, the fact that most of the transplants were performed in the 1990s, and missing data, such as details of patient characteristics and preconditioning regimens.

Hematopoietic SCT, has been performed for ATL more often in Japan than in any other country worldwide [11, 12]. We believe that it is important to report the outcomes and drawbacks of ASCT for ATL as basic data for development of new ATL therapies in the future.

## Supplementary information

Supplemental Tables
