# Supplementary material for: Long-term follow-up of patients with ATL after autologous stem cell transplantation
Source: Bone Marrow Transplant. 2022 Jan 23;57(2):323–5. doi: 10.1038/s41409-021-01412-9 (PMC8821007; doi:10.1038/s41409-021-01412-9)
Supplement: Supplementary file 1 — Supplemental Tables [file 41409_2021_1412_MOESM1_ESM.pptx]

## Slide 1
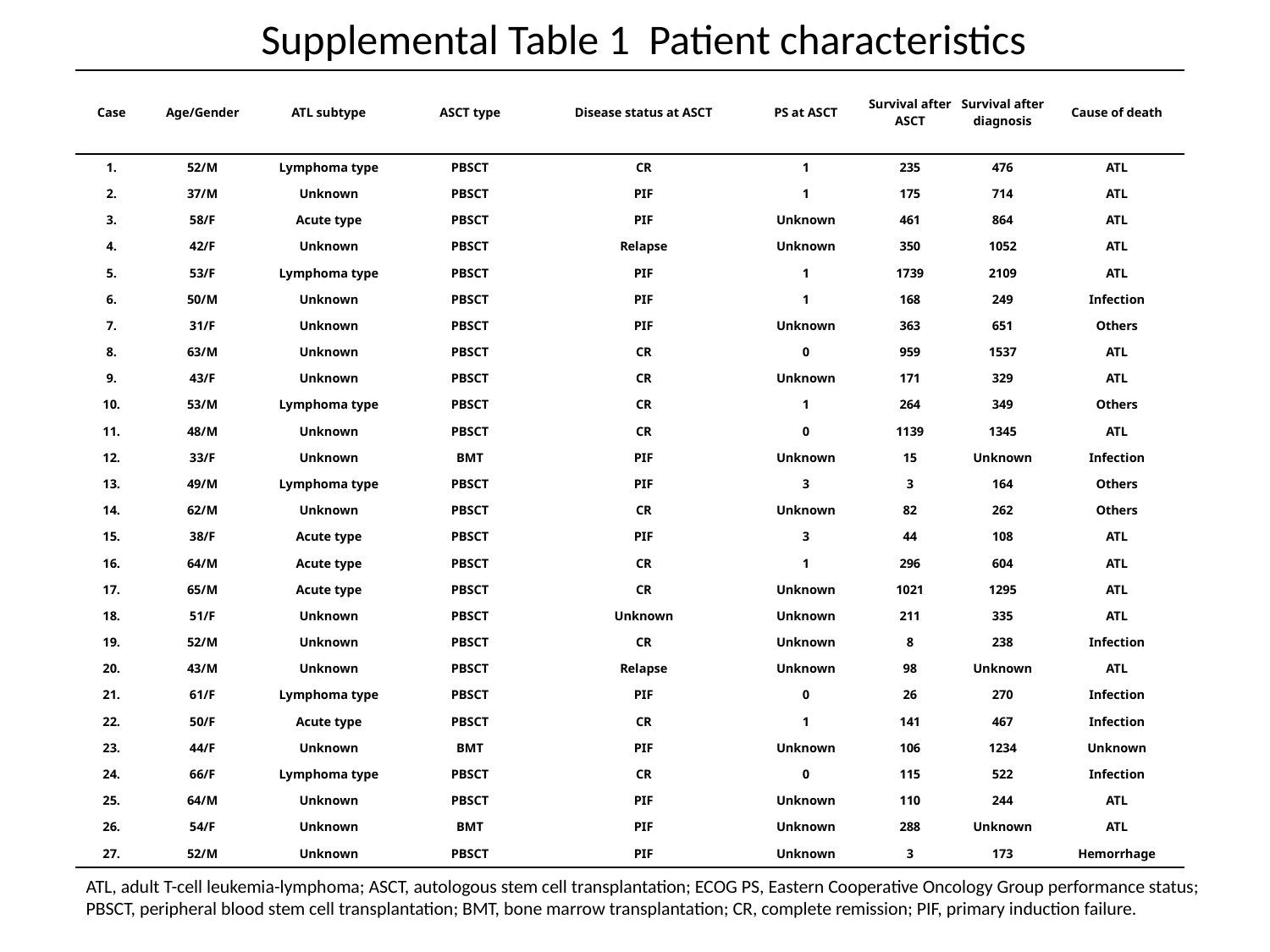

Supplemental Table 1 Patient characteristics
| Case | Age/Gender | ATL subtype | ASCT type | Disease status at ASCT | PS at ASCT | Survival after ASCT | Survival after diagnosis | Cause of death |
| --- | --- | --- | --- | --- | --- | --- | --- | --- |
| 1. | 52/M | Lymphoma type | PBSCT | CR | 1 | 235 | 476 | ATL |
| 2. | 37/M | Unknown | PBSCT | PIF | 1 | 175 | 714 | ATL |
| 3. | 58/F | Acute type | PBSCT | PIF | Unknown | 461 | 864 | ATL |
| 4. | 42/F | Unknown | PBSCT | Relapse | Unknown | 350 | 1052 | ATL |
| 5. | 53/F | Lymphoma type | PBSCT | PIF | 1 | 1739 | 2109 | ATL |
| 6. | 50/M | Unknown | PBSCT | PIF | 1 | 168 | 249 | Infection |
| 7. | 31/F | Unknown | PBSCT | PIF | Unknown | 363 | 651 | Others |
| 8. | 63/M | Unknown | PBSCT | CR | 0 | 959 | 1537 | ATL |
| 9. | 43/F | Unknown | PBSCT | CR | Unknown | 171 | 329 | ATL |
| 10. | 53/M | Lymphoma type | PBSCT | CR | 1 | 264 | 349 | Others |
| 11. | 48/M | Unknown | PBSCT | CR | 0 | 1139 | 1345 | ATL |
| 12. | 33/F | Unknown | BMT | PIF | Unknown | 15 | Unknown | Infection |
| 13. | 49/M | Lymphoma type | PBSCT | PIF | 3 | 3 | 164 | Others |
| 14. | 62/M | Unknown | PBSCT | CR | Unknown | 82 | 262 | Others |
| 15. | 38/F | Acute type | PBSCT | PIF | 3 | 44 | 108 | ATL |
| 16. | 64/M | Acute type | PBSCT | CR | 1 | 296 | 604 | ATL |
| 17. | 65/M | Acute type | PBSCT | CR | Unknown | 1021 | 1295 | ATL |
| 18. | 51/F | Unknown | PBSCT | Unknown | Unknown | 211 | 335 | ATL |
| 19. | 52/M | Unknown | PBSCT | CR | Unknown | 8 | 238 | Infection |
| 20. | 43/M | Unknown | PBSCT | Relapse | Unknown | 98 | Unknown | ATL |
| 21. | 61/F | Lymphoma type | PBSCT | PIF | 0 | 26 | 270 | Infection |
| 22. | 50/F | Acute type | PBSCT | CR | 1 | 141 | 467 | Infection |
| 23. | 44/F | Unknown | BMT | PIF | Unknown | 106 | 1234 | Unknown |
| 24. | 66/F | Lymphoma type | PBSCT | CR | 0 | 115 | 522 | Infection |
| 25. | 64/M | Unknown | PBSCT | PIF | Unknown | 110 | 244 | ATL |
| 26. | 54/F | Unknown | BMT | PIF | Unknown | 288 | Unknown | ATL |
| 27. | 52/M | Unknown | PBSCT | PIF | Unknown | 3 | 173 | Hemorrhage |
ATL, adult T-cell leukemia-lymphoma; ASCT, autologous stem cell transplantation; ECOG PS, Eastern Cooperative Oncology Group performance status;
PBSCT, peripheral blood stem cell transplantation; BMT, bone marrow transplantation; CR, complete remission; PIF, primary induction failure.

## Slide 2
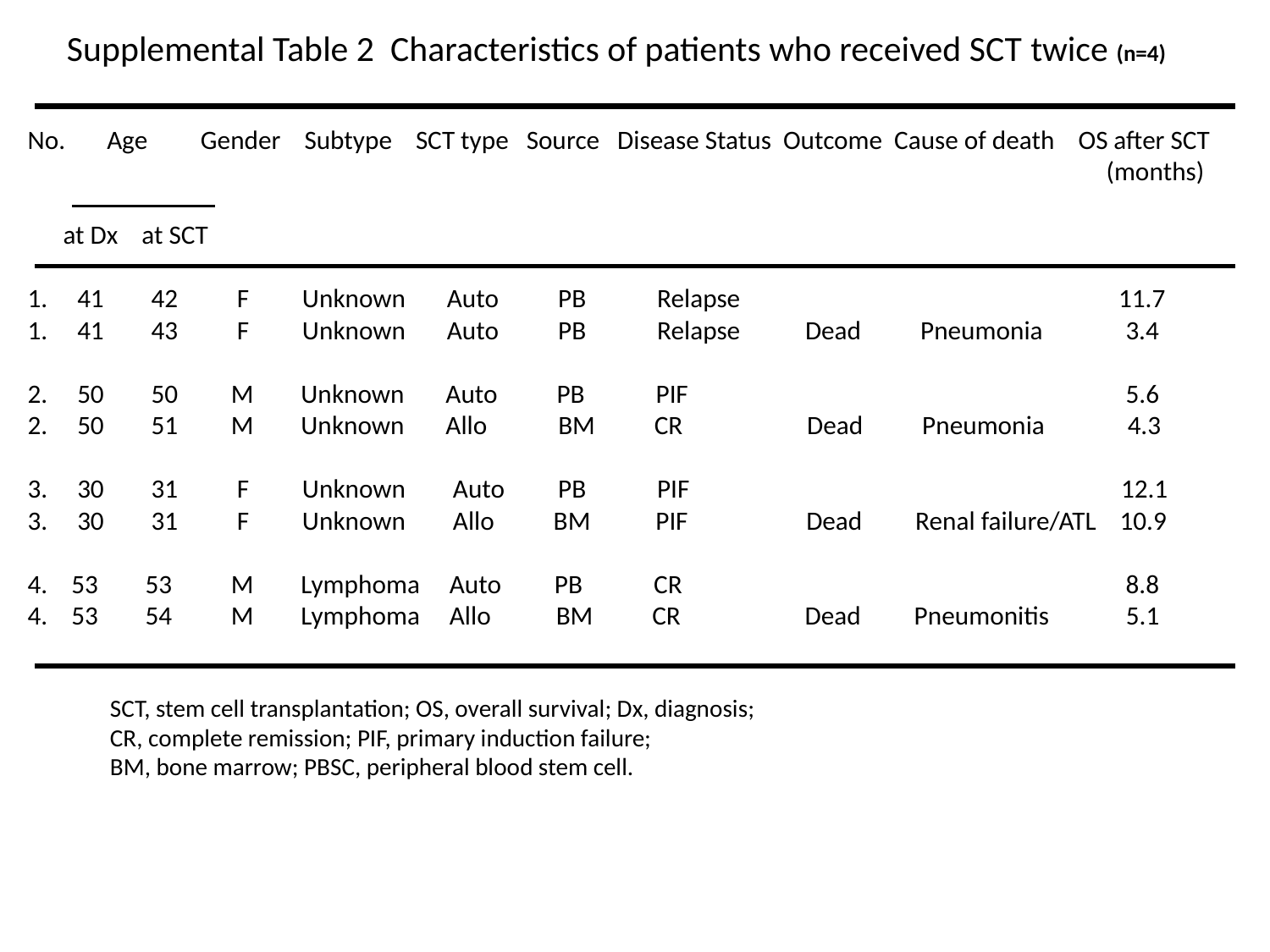

# Supplemental Table 2 Characteristics of patients who received SCT twice (n=4)
No. Age Gender Subtype SCT type Source Disease Status Outcome Cause of death OS after SCT
 (months)
 at Dx at SCT
1. 41 42 F Unknown Auto PB Relapse 11.7
1. 41 43 F Unknown Auto PB Relapse Dead Pneumonia 3.4
2. 50 50 M Unknown Auto PB PIF 5.6
2. 50 51 M Unknown Allo BM CR Dead Pneumonia 4.3
3. 30 31 F Unknown Auto PB PIF 12.1
3. 30 31 F Unknown Allo BM PIF Dead Renal failure/ATL 10.9
4. 53 53 M Lymphoma Auto PB CR 8.8
4. 53 54 M Lymphoma Allo BM CR Dead Pneumonitis 5.1
SCT, stem cell transplantation; OS, overall survival; Dx, diagnosis;
CR, complete remission; PIF, primary induction failure;
BM, bone marrow; PBSC, peripheral blood stem cell.
